# Usefulness of the CHA_2_DS_2_-VASc Score in Predicting the Outcome in Subjects Hospitalized with COVID-19—A Subanalysis of the COLOS Study

**DOI:** 10.3390/microorganisms12102060

**Published:** 2024-10-13

**Authors:** Katarzyna Resler, Pawel Lubieniecki, Tomasz Zatonski, Adrian Doroszko, Malgorzata Trocha, Marek Skarupski, Krzysztof Kujawa, Maciej Rabczynski, Edwin Kuznik, Dorota Bednarska-Chabowska, Marcin Madziarski, Tymoteusz Trocha, Janusz Sokolowski, Ewa A. Jankowska, Katarzyna Madziarska

**Affiliations:** 1 Clinical Department of Otolaryngology, Head and Neck Surgery, Wroclaw Medical University, Borowska Street 213, 50-556 Wroclaw, Poland; katarzyna.resler@umw.edu.pl (K.R.); tomasz.zatonski@umw.edu.pl (T.Z.); 2 Clinical Department of Diabetology and Internal Disease, University Hospital, Wroclaw Medical University, Borowska Street 213, 50-556 Wroclaw, Poland; malgorzata.trocha@umw.edu.pl (M.T.); maciej.rabczynski@umw.edu.pl (M.R.); edwin.kuznik@usk.wroc.pl (E.K.); dorota.bednarska-chabowska@umw.edu.pl (D.B.-C.); katarzyna.madziarska@umw.edu.pl (K.M.); 3 Clinical Department of Cardiology, 4th Military Hospital, Faculty of Medicine, Wroclaw University of Science and Technology, Weigla 5 Street, 50-981 Wroclaw, Poland; adrian.doroszko@pwr.edu.pl; 4 Faculty of Pure and Applied Mathematics, Wroclaw University of Science and Technology, 50-376 Wroclaw, Poland; marek.skarupski@pwr.edu.pl; 5 Statistical Analysis Centre, Wroclaw Medical University, K. Marcinkowski Street 2-6, 50-368 Wroclaw, Poland; krzysztof.kujawa@umw.edu.pl; 6 Clinical Department of Rheumatology and Internal Medicine, University Hospital, Borowska Street 213, 50-556 Wroclaw, Poland; marcin.madziarski@usk.wroc.pl; 7 Faculty of Medicine, Wroclaw Medical University, Borowska Street 213, 50-556 Wroclaw, Poland; tymoteusz.trocha@student.umed.wroc.pl; 8 Department of Emergency Medicine, Wroclaw Medical University, Borowska Street 213, 50-556 Wroclaw, Poland; janusz.sokolowski@umw.edu.pl; 9 Institute of Heart Diseases, Wroclaw Medical University, Borowska Street 213, 50-556 Wroclaw, Poland; ewa.jankowska@umw.edu.pl

**Keywords:** COVID-19, SARS-CoV2, CHA_2_DS_2_-VASc—score, outcomes, mortality

## Abstract

Background: The aim of this study was to see if the CHA_2_DS_2_-VASc score (Cardiac failure or dysfunction, Hypertension, Age ≥ 75 [Doubled], Diabetes, Stroke [Doubled]—Vascular disease, Age 65–74 and Sex category [Female] score) could have potential clinical relevance in predicting the outcome of hospitalization time, need for ICU hospitalization, survival time, in-hospital mortality, and mortality at 3 and 6 months after discharge home. Materials: A retrospective analysis of 2183 patients with COVID-19 hospitalized at the COVID-19 Centre of the University Hospital in Wrocław, Poland, between February 2020 and June 2021, was performed. All medical records were collected as part of the COronavirus in LOwer Silesia—the COLOS registry project. The CHA_2_DS_2_-VASc score was applied for all subjects, and the patients were observed from admission to hospital until the day of discharge or death. Further information on patient deaths was prospectively collected following the 90 and 180 days after admission. The new risk stratification derived from differences in survival curves and long-term follow-up of our patients was obtained. Primary outcomes measured included in-hospital mortality and 3-month and 6-month all-cause mortality, whereas secondary outcomes included termination of hospitalization from causes other than death (home discharges/transfer to another facility or deterioration/referral to rehabilitation) and non-fatal adverse events during hospitalization. Results: It was shown that gender had no effect on mortality. Significantly shorter hospitalization time was observed in the group of patients with low CHA_2_DS_2_-VASc scores. Among secondary outcomes, CHA_2_DS_2_-VASc score revealed predictive value in both genders for cardiogenic (5.79% vs. 0.69%; *p* < 0.0001), stroke/TIA (0.48% vs. 9.92%; *p* < 0.0001), acute heart failure (0.97% vs. 18.18%; *p* < 0.0001), pneumonia (43% vs. 63.64%; *p* < 0.0001), and acute renal failure (7.04% vs. 23.97%; *p* < 0.0001). This study points at the usefulness of the CHA_2_DS_2_-VASc score in predicting the severity of the course of COVID-19. Conclusions: Routine use of this scale in clinical practice may suggest the legitimacy of extending its application to the assessment of not only the risk of thromboembolic events in the COVID-19 cohort.

## 1. Introduction

Since the outbreak of the coronavirus disease 2019 (COVID-19) pandemic, the global healthcare system has been placed under a major challenge. The implemented preventive restrictions limited, but did not stop, the spread of the pandemic, which affected the whole world.

The rapid transmission of the disease and the number of fatalities paved the way for a search for tools that could immediately identify individuals at the highest risk and thus support clinicians in the decision-making process [1]. Initial guidelines indicated that radiological examinations should be performed, as lung lesions and acute respiratory distress syndrome were the main symptoms of SARS-CoV-2 infection [2]. Subsequent mutations of the virus extended the spectrum of symptoms and led to multi-organ failure [1,3]. As the pandemic progressed, several risk factors were identified, including advanced age and the presence of concomitant diseases, such as cardiovascular disease, chronic obstructive pulmonary disease, or diabetes [4]. One of the observed and alarming symptoms in patients with COVID-19 was the development of severe coagulopathy [5,6]. Numerous reports have described the occurrence of disseminated intravascular coagulation (DIC) in patients with SARS-CoV-2, leading commonly to fatal consequences and being a common pathophysiological denominator of target organ damage [7,8,9]. The course of the SARS-CoV-2 pandemic highlighted the need for a tool that would help stratify the risk at baseline on admission to the hospital and that would not require advanced additional diagnostics, as it could be based on the presence of comorbidities.

**Figure 1:**
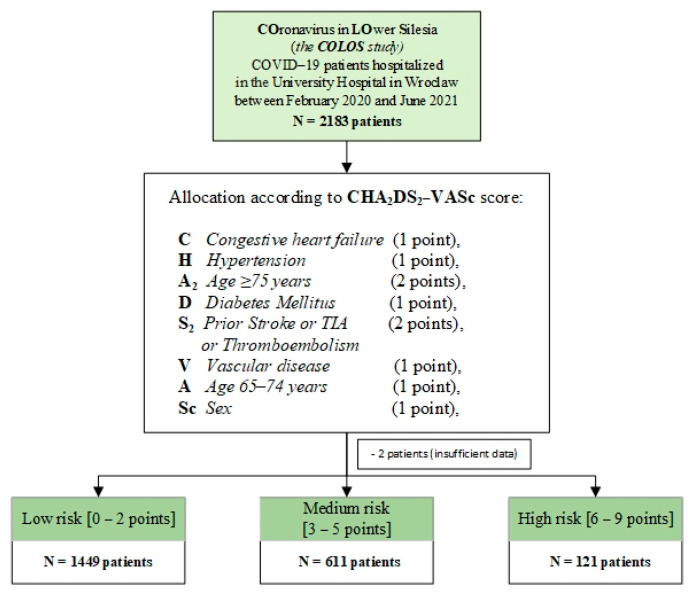
A flowchart presenting the study protocol.

Since the presence and number of comorbidities have been shown to play a key role in the development of adverse events during hospitalization, we decided to see if the CHA_2_DS_2_-VASc score (Cardiac failure or dysfunction, Hypertension, Age ≥ 75 [Doubled], Diabetes, Stroke [Doubled]—Vascular disease, Age 65–74 and Sex category [Female] score (Figure 1) [10] could have potential clinical relevance in predicting the outcome of hospitalization time, need for ICU hospitalization, survival time, in-hospital mortality, mortality at 3 and 6 months after discharge home, shock, thrombosis, myocardial infarction, heart failure, transient ischemic attack, systemic inflammatory response syndrome, and multiple organ dysfunction syndrome. The CHA_2_DS_2_-VASc score was initially introduced to stratify the risk of thromboembolic events in atrial fibrillation (AFib) [10] patients, but since acute COVID-19 is based mainly on an inflammatory response leading to a cytokine storm in some cases, as well as increased coagulation, which can consequently lead to thromboembolic events, we hypothesized that the use of this score in hospitalized COVID-19 patients on admission might allow for the prediction of some COVID-19 outcomes. In the subject literature, there are studies on the use of the CHA_2_DS_2_-VASc scale in the COVID-19 population, e.g., Arcari analyzed the clinical characteristics of patients in relation to D-dimer, CRP, and troponin [11]. Subsequently, Valente Silva analyzed the above scale in the context of a predictor of short-term mortality in patients with COVID-19 [12]. The Genc study explored the association between five thromboembolic risk scores and in-hospital events in a group of 410 patients with COVID-19 [13], whereas this is the first study to verify the utility of the CHA_2_DS_2_-VASc score in the Central European population in predicting detailed complications in a large group of 2181 patients during hospitalization of COVID-19 subjects, including other significant health complications and mortality at longer follow-up. Given these reports, we aimed to test the utility of the CHA_2_DS_2_-VASc score in a group of patients included in the COLOS study in patients with COVID-19.

## 2. Materials and Methods

### 2.1. Study Design and Population

In the current study, a retrospective analysis of 2183 patients with COVID-19 hospitalized at the COVID-19 Centre of the University Hospital in Wrocław, Poland, between February 2020 and June 2021, was performed. In all patients, rigorously following the protocol published by the World Health Organization (WHO), SARS-CoV-2 infection was confirmed by reverse transcription–polymerase chain reaction (RT-PCR) in nasopharyngeal swabs. All medical records were collected as part of the COronavirus in LOwer Silesia—the COLOS registry project. All studies were conducted in accordance with relevant guidelines and regulations. The study protocol has been accepted by the Institutional Review Board and Ethics Committee at the Wroclaw Medical University, Wroclaw, Poland (No. KB-444/2021). Informed consent was obtained from all study participants. All available medical data were fully anonymized and then analyzed retrospectively. The CHA_2_DS_2_-VASc score was calculated according to the following variables: congestive heart failure (1 point), hypertension (1 point), age ≥ 75 years (2 points), diabetes mellitus (1 point), prior stroke or TIA or thromboembolism (2 points), vascular disease (1 point), age 65–74 years (1 point), and sex (1 point). Diabetes mellitus was diagnosed according to the criteria of the American and Polish Diabetes Societies. The data reviewed were based on demographic information, clinical characteristics, mechanical ventilation, smoking, comorbidities, previous medications, laboratory results, and hospitalization history. Based on the calculated score, subjects were assigned to one of three groups—low risk, 0 to 2 points; intermediate risk, 3 to 5 points; and high risk, 6 to 9 points. Figure 1 shows the study protocol.

### 2.2. Clinical Follow-Up and Outcomes

The time interval analyzed included observation from the time of admission to the hospital until the day of discharge or death. Further information on patient deaths was prospectively collected following 90 and 180 days after admission. Outcomes measured included in-hospital mortality, 3-month and 6-month all-cause mortality, and termination of hospitalization from causes other than death (home discharges/transfer to another facility or deterioration/referral to rehabilitation).

The analyzed data included demographic information, clinical characteristics, breathing support, smoking, comorbidities, home medication, laboratory results, and the course of hospitalization, including adverse clinical events, such as shock [14], pulmonary embolism [15], deep-vein thrombosis [15], myocardial infarction [16], myocardial injury (defined as more than a 3-fold increase in serum troponin levels above the upper range limit), acute heart failure [17], stroke/TIA [18], pneumonia, complete respiratory failure [19], systemic inflammatory response syndrome (SIRS) [20], sepsis [21], acute kidney injury [22], acute liver dysfunction (serum bliribubin > 2.0 mg/dL and INR > 1.5), multiple organ dysfunction syndrome (MODS) [20], and bleeding (macroscopic or confirmed by imaging diagnostics).

### 2.3. Study Groups—CHA_2_DS_2_-VASc Score Stratification

The entire study group (2181 patients) was divided into three groups according to the CHA_2_DS_2_-VASc score obtained on hospital admission. The following seven variables reported during the medical history at admission were included in the calculations: congestive heart failure, hypertension, diabetes mellitus, prior stroke or TIA or thromboembolism, vascular disease, sex, and age (in two views). Once the CHA_2_DS_2_-VASc scores were calculated, patients were assigned to the specific groups according to their scores as follows: the low-risk subgroup, ≤2 pts; the medium-risk subgroup, 3 to 5 pts; and the high-risk subgroup, ≥6 points.

The scoring risk strata for the respective groups were obtained by the log-rank statistical analysis against the survival curves of all possible CHA_2_DS_2_-VASc intervals, and a risk score was calculated for each in Table 1. The log-rank test is used to select the best points of scale intersection, distinguishing the best-differentiated subgroups from each other. The best results were obtained for the range shown above. The best results were obtained for the log rank (as represented by the two highest log-rank values) shown in Supplementary Table S1. As in-hospital mortality and all-cause mortality were available as right-censored data, time-dependent ROC analysis with inverse probability of censoring (IPCW) was used to estimate them. The time-dependent area under the curve (AUC) was used to assess the CHA_2_DS_2_-VASc score.

### 2.4. Statistical Analysis

Descriptive data are presented as numbers and percentages for categorical variables, as the mean with a standard deviation range (minimum–maximum), and as the number of non-missing values for numerical variables. An omnibus test chi-square test was used for categorical variables with more than 5 expected cases in each group, whereas Fisher’s exact test was used for cases with fewer cell counts. If needed, the post hoc test was the same as the omnibus test but performed for subgroups using Bonferroni correction.

Due to the sample size being large enough for the appropriateness of asymptotic results (i.e., irrespective from the data distribution), ANOVA was performed for the comparison of continuous variable means. As the variances differed between risk strata, Welch’s correction was used. Post hoc analysis for continuous variables was performed using the Games–Howell test with Tukey correction.

The primary outcomes, i.e., in-hospital mortality and all-cause mortality data, were available as right-censored data; thus, a time-dependent ROC analysis with inverse probability of censoring weighting (IPCW) estimation was performed for those variables. The CHA_2_DS_2_-VASc score effect was assessed through the time-dependent area under the curve (AUC). A Cox proportional hazard model was used to analyze the hazard ratio (HR) in relation to the CHA_2_DS_2_-VASc score, its components, and risk strata. The proportional hazard assumption for Cox regression was verified using the Grambsch–Therneau test. A log-rank test was used to confirm differences in survival curves between risk strata.

For the secondary outcomes, due to their dichotomic nature, a logistic regression model was fitted. Classical ROC analysis was performed, and the AUC measure was used for assessing predictive capabilities. The odds ratio (OR) was reported as the effect size for the influence of the CHA_2_DS_2_-VASc score, its components, and risk strata. In the case of the scoring risk strata for the study groups, the proportional hazards assumption was verified using the Grambsch–Therneau test. When analyzing the hazard ratio (HR) of the CHA_2_DS_2_-VASc scale, its components, and the risk strata, the Cox proportional hazards model was used.

All statistical analyses were performed with R version 4.0.4 using packages time–ROC, pROC [23], survival [24], coin [25], and odds ratio [26]. A significance level of 0.05 was selected for all statistical analyses.

## 3. Results

### 3.1. Patients’ Baseline Characteristics

The analyzed patient group consisted of 2181 subjects (male = 1081, 49%) at a mean age of 60.06 ± 18.84 years who were then allocated on the basis of their CHA_2_DS_2_-VASc score to the low-risk stratum, n = 1449 patients (male = 722, 49.83%; age 52.05 ± 17.18), the medium-risk stratum, n = 611 patients (male = 292, 47.79%; age 75.29 ± 9.95), and the high-risk stratum, n = 121 groups (male = 67, 55.37%; age 78.94 ± 8.62), respectively. In the study population, a higher risk, as assessed by CHA_2_DS_2_-VASc, was associated with more comorbidities and more advanced age (52.05 vs. 75.3 vs. 78.9, for the low-, medium-, and high-risk groups, respectively). The baseline characteristics of the study group are summarized in Table 1. In addition, the prevalence of cigarette smoking was significantly higher in the medium- and high-risk subgroups. Consecutively, hypertension, diabetes, dyslipidemia, atrial fibrillation/flutter, myocardial infarction, heart failure, peripheral artery disease, hemodialysis, transient ischemic attack, chronic kidney disease, and chronic obstructive pulmonary disease were significantly higher in the high-risk than the low-risk CHA_2_DS_2_-VASc stratum.

In the high-risk stratum, differences were observed in the treatment administered before hospitalization. Individuals in this group were more likely to receive cardiovascular drugs, including mineralocorticoid receptor antagonists (MRAs), calcium channel blockers, b-blockers, loop diuretics, statins, P2Y12 inhibitors, new oral anticoagulants (NOACs), and insulin. Notably, no differences regarding the drugs affecting the immune response, such as steroids and immunosuppressants between particular risk strata in the pre-hospital period, were observed. All the data on treatment administered before hospitalization are shown in Supplementary Table S2.

Chest discomfort with wheezing on admission was significantly more common in the high-risk group. In the medium-risk group with respect to the low-risk group, cough, olfactory disturbances, cramps, pulmonary congestion, peripheral edema, PP, and SBP were significantly more frequent, and the low-risk group achieved higher SpO_2_ values. There were no significant differences in the prevalence of other symptoms among the three risk strata CHA_2_DS_2_-VASc scores. Patient-reported symptoms, vital signs, and abnormalities measured during the physical examination at hospital admission are summarized in Table 2.

### 3.2. Laboratory Assays

Supplementary Table S3 presents detailed characteristics of the laboratory parameters measured on admission and during hospitalization. At admission, the high-risk group had the lowest hemoglobin and procalcitonin levels. At the same time, this cohort had significantly higher potassium levels. The low-risk group presented significantly better renal function parameters at admission, i.e., significantly lower urea and creatinine levels and significantly higher eGFR. At the same time, abnormalities in INR and APTT were much more frequently observed in this cohort. With respect to the highest risk group, it presented significantly higher levels of total protein and ALT. Those in the high-risk stratum initially had the highest mean levels of biomarkers of cardiac injury (BNP, NT-proBNP, and troponin). No significant differences were observed regarding TSH and peripheral thyroid hormones in the subgroups analyzed.

### 3.3. Drug Therapy and Applied Treatment during Hospitalization

#### 3.3.1. Drug Therapy

In general, there were no differences in the treatment used during hospitalization among the three CHA_2_DS_2_-VASc risk strata. The only exception was the frequency of catecholamines, which was slightly higher in the medium-risk group, as well as antibiotic therapy, which was rising with the CHA_2_DS_2_-VASc score. Patients in the low-risk stratum received this treatment significantly less often than those in the intermediate- and high-risk strata. Data on the overall management of study participants are shown in Table 3.

#### 3.3.2. Treatment Procedures

The intermediate-risk group was characterized by more frequent use of catecholamines. In contrast, we observed a more frequent need for coronarography in the high-risk group. On the other hand, patients in the low-risk stratum were statistically more likely not to require respiratory support and presented higher SpO_2_. The treatments and procedures used are shown in Table 4.

### 3.4. Clinical Outcome

Table 5 shows the data on the associations between the CHA_2_DS_2_-VASc risk stratum and mortality. Significant differences were found in terms of in-hospital mortality, followed by 3-month and 6-month mortality, which were the highest in the CHA_2_DS_2_-VASc high-risk stratum, reaching 35.54%, 55.0%, and 75.7.9%, respectively. Notably, in the medium-risk stratum, mortality rates reached 26.19%, 42.76%, and 64.65%, while in the low-risk stratum, they were 8.49%, 16.48%, and 39.27%, respectively.

#### 3.4.1. CHA_2_DS_2_-VASc Score Results and Mortality

**Figure 2:**
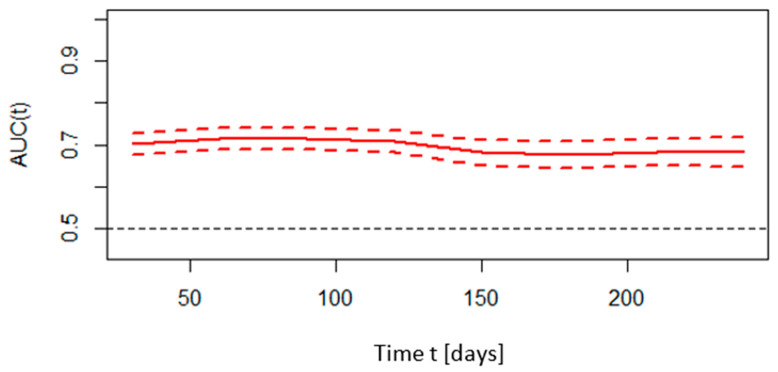
The changes in the area under the receiver operating characteristic curve (AUC) for CHA_2_DS_2_-VASc predictive abilities of all-cause death in relation to time. Explanations: AUC(t)—area under receiver operating curve as a function of time (solid line); dashed lines determine 95% confidence intervals.

A time ROC analysis was performed to assess the predictive ability of the CHA_2_DS_2_-VASc scale of deaths at time *t* from hospital admission. All causes of death were considered in the analysis. The graph below shows the predictive abilities expressed as the area under the ROC curve versus time, along with the confidence intervals for this plot. For the CHA_2_DS_2_-VASc scale, the time-dependent AUC in predicting all-cause mortality in the period extending from the day of hospital admission to 240 days after the initial diagnosis was above 60. This is shown in Figure 2.

**Figure 3:**
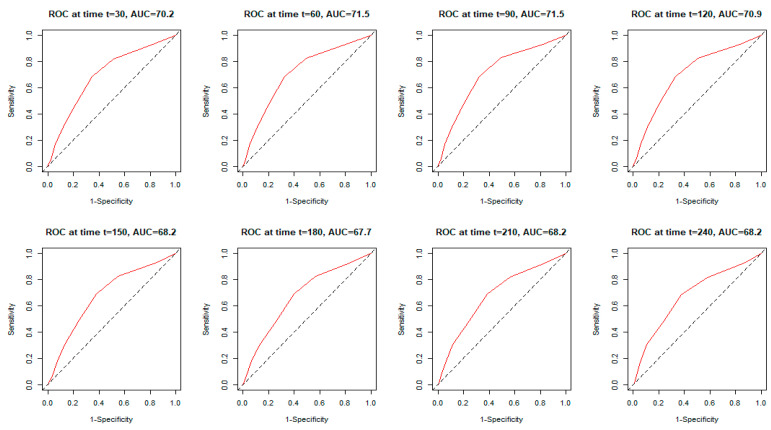
Time-dependent receiver operating characteristic curves (time–ROC) for the CHA_2_DS_2_-VASc score in predicting total mortality, determined every 30 days in the analyzed follow-up period.

Figure 3 shows the monthly time-dependent receiver operating characteristics (time–ROC) related to the CHA_2_DS_2_-VASc score. During the first half of the controlled time period, the CHA_2_DS_2_-VASc score remained at a similar level, allowing mortality to be predicted with an AUC in the range of 70.2 to 71.5. Beyond 120 days, the AUC value remained in the range of 67.7 to 68.2.

#### 3.4.2. CHA_2_DS_2_-VASc Score and Secondary Outcome

Table 6 shows all the clinical non-fatal events and hospitalization outcomes. Hospitalization of patients in the low-risk stratum lasted significantly shorter, and a large percentage of these patients were discharged home in full recovery. Patients in the high-risk stratum were more prone to develop acute heart failure, myocardial infarction, and cardiogenic shock in the course of hospitalization. Pneumonia and acute renal failure were more frequently observed in the low-risk group, while acute liver failure was more often noted in the medium-risk group. An increase in the CHA_2_DS_2_-VASc score did not raise the incidence of total or gastrointestinal bleeding. It is worth noting that there were no significant differences in the incidence of multiple organ dysfunction syndrome (MODS). There were also no differences in relation to the incidence of thromboembolic events, including both deep vein thrombosis and pulmonary embolism.

## 4. Discussion

This is the first study to assume a new risk stratification derived from differences in survival curves and long-term follow-up of our patients. Quite quickly, it became apparent that during the pandemic, it was necessary to identify risk factors and tools that were needed to qualify patients into risk strata and thus be able to predict adverse events, like ICU hospitalization or death. The Brescia-COVID Respiratory Severity Scale (BCRSS) is based on the results of the patient’s examination along with an assessment of the need for respiratory support (non-invasive ventilation, intubation, pronation), which determines further therapeutic decisions. The scale simplifies the clinical summary of the patient’s condition and allows clinicians to compare patients among themselves and track the patient’s respiratory severity over time [27]. Meanwhile, several more scales predicting unfavorable COVID-19 outcomes have been presented, including the VACO index [28] and the PRIEST score [29]. These models, however, are based on a range of clinical and laboratory data, which hinders their wider application in daily clinical practice. It was observed that hypertension, diabetes mellitus, cerebrovascular disease, coronary artery disease, renal dysfunction, and chronic obstructive pulmonary disease were more often associated with worse clinical outcomes in patients with COVID-19 [30]. Therefore, there was a need to invent a more comprehensive endpoint risk assessment tool.

The CHA_2_DS_2_-VASc scale was originally developed to assess the risk of thromboembolic complications in patients with atrial fibrillation and to identify patients with atrial fibrillation who require anticoagulant therapy [10]. Given the incidence of thromboembolic complications in COVID-19 patients [6], it seems appropriate to verify the usefulness of this scale for assessing endpoints, such as the need for intensive care unit hospitalization and in-hospital mortality, at 3- and 6-month follow-up. Previous studies [30,31,32,33,34,35], with the use of the scale in the group of patients with COVID-19, adopted the original division of points into risk strata. For our patient cohort, after analyzing all possible CHA_2_DS_2_-VASc ranges, we selected the best possible risk stratification for differences in Kaplan–Meier survival curves. In our study, among secondary outcomes, the CHA_2_DS_2_-VASc score revealed predictive value in both genders for cardiogenic (*p* < 0.0001), stroke/TIA (*p* < 0.0001), acute heart failure (*p* < 0.0001), pneumonia (*p* < 0.0001), and acute renal failure (*p* < 0.0001). In earlier discussions of the scale itself, the validity of awarding points for gender was raised, indicating by definition the placement of women in the higher-risk stratum [36]. The actual risk from being female appears to become more consequential as the number of additional risk factors increases with age, as older women have a significantly higher risk of stroke than their male counterparts [37]. Observations made in a group of COVID-19 patients indicated male gender was a risk factor for adverse events [38]. Moreover, there was even a modification of the scale, swapping gender in the scoring [35]. Katkat et al. [34] do not confirm these observations, indicating that gender did not have an influence on mortality. This is in line with our observations, where statistical calculations showed no significant change in the final effects obtained.

Based on the concurrent observations of higher mortality among COVID-19 patients with higher CHA_2_DS_2_-VASc scores, we share the opinion of Vedat Çiçek and co-authors [39] that this scale can be useful for predicting in-hospital mortality as well as 3- and 6-month follow-up in these patients. At the same time, the easy-to-calculate score will help us achieve early identification of high-risk COVID-19 patients during their hospitalization. Cetinkal and co-authors [35] using modified CHA_2_DS_2_-VASc indicate similarly to us that as the scale score increases, the risk of patient deterioration and the need for transfer to another ward/hospital increases. Our observations also indicate significantly shorter hospitalization time in the group of patients with low CHA_2_DS_2_-VASc scores.

COVID-19-induced heart disease, described as ‘acute cardiac injury’, occurs in more than 20% of patients and appears to be associated with increased mortality [40]. In our observation, the percentage of shock in patients was comparable; however, cardiogenic shock was significantly more frequent in the medium- and high-risk groups, and septic shock was significantly more frequent in the medium-risk group with respect to the low-risk group.

We share the opinion of Sonsoz and co-authors [41] that the scale is suitable for predicting the risk of stroke and acute heart failure. The authors prove that as the score of the modified (replacement of points for gender) CHA_2_DS_2_-VASc scale increases, the risk of an adverse cardiac event increases. The group notes, however, that based on ROC analysis comparing the predictive accuracy of M-CHA_2_DS_2_-VASc and CHA_2_DS_2_-VASc and based on a 95% CI, the areas under the curve (AUCs) for M-CHA_2_DS_2_-VASc, C were 0.80 and 0.79, respectively, with *p* < 0.001. In view of the above, one can assume the coincidence of the presented observations with ours. The authors also note that although the male gender was found to be associated with an increased risk of in-hospital mortality in patients with COVID-19, it was not associated with acute cardiac injury, and a similar view is shared by the authors of another study by Huayan et al. [42]. This is in line with our assumptions and the lack of modification of the scale with respect to gender.

Sadeghmousavi’s study [43] identifies stroke as one of the complications of COVID-19. However, the authors of the meta-analysis [44] show that the combined incidence of ischemic stroke in COVID-19 was 2%. Our results show a significant increase in the risk of stroke, TIA, and cognitive impairment with belonging to a higher-risk group. A paper by Chiara Di Mitri [45] reporting on outcomes in the early stages of the COVID-19 pandemic found no statistical difference in in-hospital mortality resulting from pneumonia between patients with and without COVID-19. Moreover, they point to a higher burden of the disease in the patient population without COVID-19, which is in contrast to our observations where the risk of pneumonia increases with a higher CHA_2_DS_2_-VASc scale score. Considering the causes of acute renal failure (AKI), such as endothelial dysfunction, hypercoagulability, rhabdomyolysis, and sepsis, as well as reduced oxygen delivery to the kidneys [46], it seems reasonable to observe an increase in the incidence of AKI with an increase in the CHA_2_DS_2_-VASc score, which is also positively correlated with the incidence of sepsis and circulatory disorders. Similar conclusions are indicated by a meta-analysis conducted by Lin [47], highlighting severe COVID-19 as an independent risk factor for AKI.

## 5. Conclusions

This study points out the usefulness of the CHA_2_DS_2_-VASc score in predicting the severity of the course of COVID-19, the need and intensity of required oxygen support, in-hospital mortality, and all-cause mortality in the six-month post-discharge observation period. A higher CHA_2_DS_2_-VASc score was associated with a higher incidence of cardiovascular complications during the hospitalization period but also a greater incidence of pneumonia, sepsis, acute kidney and liver injury, cognitive function impairment, and bleeding. Routine use of this scale in clinical practice may suggest the legitimacy of extending its application to the assessment of not only the risk of thromboembolic events in the COVID-19 cohort. As it reflects multimorbidity, it may be considered to extend its use in stratifying the risk of complications in COVID-19 populations, regardless of the presence of atrial fibrillation.

## 6. Limitations

The clinical outcome may be affected by a single-center registry result and retrospective analysis of the results. Moreover, some clinical data provided at the admission to the hospital and baseline laboratory assays conducted during the hospital stay may be incomplete, causing difficulty in the proper interpretation of the results. Data were collected at the beginning of the pandemic when there was still one serotype of the virus and a vaccine did not yet exist. In view of this, we do not have another cohort to assess reproducibility.

## Figures and Tables

**Table 1:** Baseline demographics and clinical characteristics.

Variables, Units (N)	Low Risk	Medium Risk	High Risk	OMNIBUS *p* Value	*p* Value (for Post Hoc Analysis)
	Demographics				
**Age, years** **mean ± SD** **min–max** **(N = 2181)**	**52.05 ± 17.18**	**75.29 ± 9.95**	**78.94 ± 8.62**	<0.0001	<0.0001 ^a,b^
	17–99	65–100	47–94		0.0002 ^c^
	(1449)	(611)	(121)		
Age ≥ 65 years n/N (% of risk category) (N = 2181)	379/1449 (26.16%)	550/611 (90.02%)	116/121 (95.87%)	<0.0001	<0.0001 ^a,b^
					0.1806 ^c^
Male gender n/N (% of risk category) (N = 2181)	722/1449 (49.83%)	292/611 (47.79%)	67/121 (55.37%)	0.295	N/A
BMI, kg/m^2^ mean ± SD min–max (N = 554)	28.33 ± 5.28	28.37 ± 5.4	29.12 ± 4.61	0.7002	N/A
	15.36–49.38	16.41–48.21	22.2–38.97		
	(398)	(129)	(27)		
Obesity (BMI ≥ 30 kg/m^2^) n/N (% of risk category) (N = 554)	131/398 (32.91%)	49/129 (37.98%)	11/27 (40.74%)	0.4924	N/A
Cigarette smoking n/N (% of risk category) never/previous/current (N = 2177)	1358/1448 (93.78%)	529/609 (86.86%)	98/120 (81.67%)	<0.0001	<0.0001 ^a,b^
	55/1448 (3.8%)	50/609 (8.21%)	12/120 (10.0%)		0.6624 ^c^
	35/1448 (2.42%)	30/609 (4.93%)	10/120 (8.33%)		
	Comorbidities				
Hypertension n/N (% of risk category) (N = 2181)	372/1449 (25.67%)	531/611 (86.91%)	116/121 (95.87%)	<0.0001	<0.0001 ^a,b^
					0.0237 ^c^
DM n/N (% of risk category) (N = 2181)	115/1449 (7.94%)	272/611 (44.68%)	84/121 (69.42%)	<0.0001	<0.0001 ^a,b,c^
Dyslipidemia n/N (% of risk category) (N = 824)	268/406 (66.01%)	270/333 (81.08%)	72/85 (84.71%)	<0.0001	<0.0001 ^a^
					0.0033 ^b^
					1.0 ^c^
AF/AFL n/N (% of risk category) (N = 2181)	47/1449 (3.24%)	186/611 (30.44%)	57/121 (47.11%)	<0.0001	<0.0001 ^a,b^
					0.0017 ^c^
Previous coronary revascularization n/N (% of risk category) (N = 2181)	10/1449 (0.69%)	91/611 (14.89%)	52/121 (42.98%)	<0.0001	<0.0001 ^a,b,c^
Previous MI n/N (% of risk category) (N = 2181)	22/1449 (1.52%)	112/611 (18.33%)	57/121 (47.11%)	<0.0001	<0.0001 ^a,b,c^
HF n/N (% of risk category) (N = 2181)	17/1449 (1.17%)	155/611 (25.37%)	82/121 (67.77%)	<0.0001	<0.0001^a,b,c^
Moderate or severe valvular heart disease or previous valve heart surgery n/N (% of risk category) (N = 2181)	21/1449 (1.45%)	51/611 (8.35%)	24/121 (19.83%)	<0.0001	<0.0001 ^a,b^
					0.0008 ^c^
PAD n/N (% of risk category) (N = 2181)	11/1449 (0.76%)	58/611 (9.49%)	30/121 (24.79%)	<0.0001	<0.0001 ^a,b,c^
Previous stroke/TIA n/N (% of risk category) (N = 2181)	8/1449 (0.55%)	78/611 (12.77%)	77/121 (63.64%)	<0.0001	<0.0001 ^a,b,c^
CKD n/N (% of risk category) (N = 2181)	73/1449 (5.04%)	111/611 (18.17%)	47/121 (38.84%)	<0.0001	<0.0001 ^a,b,c^
Hemodialysis n/N (% of risk category) (N = 2181)	20/1449 (1.38%)	26/611 (4.26%)	12/121 (9.92%)	<0.0001	0.0004 ^a^
					<0.0001 ^b^
					0.065 ^c^
Asthma n/N (% of risk category) (N = 2181)	54/1449 (3.73%)	27/611 (4.42%)	4/121 (3.31%)	0.7231	N/A
COPD n/N (% of risk category) (N = 2181)	21/1449 (1.45%)	42/611 (6.87%)	12/121 (9.92%)	<0.0001	<0.0001 ^a,b^
					0.7598 ^c^
Thyroid disease, none/hypothyroidism/ hyperthyroidism n/N (% of risk category) (N = 2181)	1316/1449 (90.82%)	535/611 (87.56%)	102/121 (84.3%)	0.04	0.193 ^a^
	122/1449 (8.42%)	68/611 (11.13%)	17/121 (14.05%)		0.1307 ^b^
	11/1449 (0.76%)	8/611 (1.31%)	2/121 (1.65%)		1.0 ^c^

Continuous variables are presented as mean ± SD, range (minimum–maximum), and the number of non-missing values. Categorized variables are presented as a number with a percentage. Information about the numbers with valid values is provided in the left column. Abbreviations: N—valid measurements, n—number of patients with parameters above the cut-off point, SD—standard deviation, BMI—body mass index, DM—diabetes mellitus, AF/AFL—atrial fibrillation/flutter, MI—myocardial infarction, HF—heart failure, PAD—peripheral artery disease, TIA—transient ischemic attack, CKD—chronic kidney disease, COPD—chronic obstructive pulmonary disease, N/A—non-applicable, OMNIBUS—overall Welch ANOVA or chi-square test, ^a^—low risk vs. medium risk, ^b^—low risk vs. high risk, ^c^—medium risk vs. high risk, W—Welch ANOVA, X—chi-square test.

**Table 2:** Patient-reported symptoms, vital signs, and abnormalities measured during physical examination at hospital admission in the studied cohort after CHA_2_DS_2_-VASc risk stratification.

Variables, Units (N)	Low Risk	Medium Risk	High Risk	OMNIBUS *p* Value	*p* Value (for Post Hoc Analysis)
	Patient-Reported Symptoms				
**Cough** **n/N (% of risk category)** **(N = 2181)**	**482/1449 (33.26%)**	**138/611 (22.59%)**	**27/121 (22.31%)**	<0.0001	<0.0001 ^a^
					0.0532 ^b^
					1.0 ^c^
Dyspnea n/N (% of risk category) (N = 2181)	604/1449 (41.68%)	262/611 (42.88%)	54/121 (44.63%)	0.7533	N/A
Chest pain n/N (% of risk category) (N = 2181)	110/1449 (7.59%)	36/611 (5.89%)	17/121 (14.05%)	0.0074	0.6027 ^a^
					0.0595 ^b^
					0.0089 ^c^
Hemoptysis n/N (% of risk category) (N = 2181)	10/1449 (0.69%)	3/611 (0.49%)	2/121 (1.65%)	0.2843	N/A
Smell dysfunction n/N (% of risk category) (N = 2181)	63/1449 (4.34%)	11/611 (1.8%)	2/121 (1.65%)	0.0071	0.0121 ^a^
					0.6906 ^b^
					1.0 ^c^
Taste dysfunction n/N (% of risk category) (N = 2181)	52/1449 (3.59%)	12/611 (1.96%)	2/121 (1.65%)	0.1157	N/A
Abdominal pain n/N (% of risk category) (N = 2181)	107/1449 (7.38%)	31/611 (5.07%)	8/121 (6.61%)	0.1592	N/A
Diarrhea n/N (% of risk category) (N = 2181)	74/1449 (5.11%)	46/611 (7.53%)	7/121 (5.79%)	0.1004	N/A
Nausea and/or vomiting n/N (% of risk category) (N = 2181)	60/1449 (4.14%)	31/611 (5.07%)	7/121 (5.76%)	0.5042	N/A
	Measured vital signs				
Body temperature °C mean ± SD min–max (N = 1184)	37.07 ± 0.89	36.9 ± 0.88	36.98 ± 0.88	0.0141	0.01 ^a^
	34.4–40.5	35.0–40.0	35.9–40.0		0.667 ^b^
	(818)	(301)	(65)		0.799 ^c^
Heart rate beats/minute mean ± SD min–max (N = 1670)	86.17 ± 15.59	84.58 ± 17.31	85.31 ± 18.82	0.2136	N/A
	48–160	36–150	47–170		
	(1063)	(499)	(108)		
Respiratory rate breaths/minute mean ± SD min–max (N = 317)	18.43 ± 5.64	18.6 ± 5.4	19.35 ± 7.84	0.8489	N/A
	12–50	12–50	12–50		
	(207)	(87)	(23)		
PP mean ± SD min–max (N = 1658)	52.26 ± 14.97	57.46 ± 18.98	59.96 ± 19.15	<0.0001	<0.0001 ^a^
	11–115	15–136	20–120		0.0002 ^b^
	(1046)	(500)	(112)		0.425 ^c^
SBP mmHg mean ± SD min–max (N = 1667)	130.42 ± 20.56	134.27 ± 25.7	137.25 ± 27.21	0.0012	0.009 ^a^
	60–237	50–240	85–270		0.029 ^b^
	(1050)	(505)	(112)		0.54 ^c^
DBP mmHg mean ± SD min–max (N = 1659)	78.35 ± 12.39	77.49 ± 14.9	77.29 ± 14.75	0.4505	N/A
	40–150	40–157	50–150		
	(1047)	(500)	(112)		
SpO_2_ on room air, % (FiO_2_ = 21%) mean ± SD min–max (N = 1260)	92.54 ± 7.44	90.37 ± 9.19	90.0 ± 8.27	<0.0001	0.0004 ^a^
	48–100	50–100	60–100		0.022 ^b^
	(836)	(340)	(84)		0.93 ^c^
	Abnormalities detected during physical examination				
Cracles n/N (% of risk category) (N = 2181)	161/1449 (11.11%)	127/611 (20.79%)	31/121 (25.62%)	<0.0001	<0.0001 ^a,b^
					0.8675 ^c^
Wheezing n/N (% of risk category) (N = 2181)	101/1449 (6.97%)	88/611 (14.4%)	30/121 (24.79%)	<0.0001	<0.0001 ^a,b^
					0.0205 ^c^
Pulmonary congestion n/N (% of risk category) (N = 2181)	192/1449 (13.25%)	140/611 (22.91%)	34/121 (28.1%)	<0.0001	<0.0001 ^a,b^
					0.8043 ^c^
Peripheral edema n/N (% of risk category) (N = 2181)	88/1449 (6.07%)	80/611 (13.09%)	21/121 (17.36%)	<0.0001	<0.0001 ^a,b^
					0.817 ^c^

Continuous variables are presented as mean ± SD, range (minimum–maximum), and the number of non-missing values. Categorized variables are presented as a number with a percentage. Information about the numbers with valid values is provided in the left column. Abbreviations: N—valid measurements. n—number of patients with parameters above the cut-off point. SD—standard deviation. N/A—non-applicable. ^a^—low risk vs. medium risk. ^b^—low risk vs. high risk. ^c^—medium risk vs. high risk.

**Table 3:** Therapies applied during hospitalization in the studied cohort.

Variables, Units (N)	Low Risk	Medium Risk	High Risk	Overall Chi-Square Test *p* Value	*p* Value (for Post Hoc Analysis)
	Applied Treatment and Procedures				
**Systemic corticosteroid n/N** **(% of risk category)** **(N = 2181)**	**715/1449 (49.34%)**	**325/611 (53.19%)**	**56/121 (46.28%)**	**0.1871**	N/A
Convalescent plasma n/N (% of risk category) (N = 2181)	158/1449 (10.9%)	69/611 (11.29%)	12/121 (9.92%)	0.2008	N/A
Tocilizumab n/N (% of risk category) (N = 2181)	21/1449 (1.45%)	4/611 (0.65%)	0/121 (0%)	0.1955	N/A
Remdesivir n/N (% of risk category) (N = 2181)	238/1449 (16.43%)	87/611 (14.24%)	18/121 (14.88%)	0.4449	N/A
Antibiotic n/N (% of risk category) (N = 2181)	756/1449 (52.17%)	398/611 (65.14%)	86/121 (71.07%)	<0.0001	<0.0001 ^a^
					0.0003 ^b^
					0.7441 ^c^

Categorized variables are presented as a number with a percentage. Information about the numbers with valid values is provided in the left column. Abbreviations: N—valid measurements. n—number of patients with parameters above the cut-off point. N/A—non-applicable. ^a^—low risk vs. medium risk. ^b^—low risk vs. high risk. ^c^—medium risk vs. high risk.

**Table 4:** Applied treatment and procedures.

Variables, Units (N)	Low Risk	Medium Risk	High Risk	OMNIBUS *p* Value	*p* Value (for Post Hoc Analysis)
	Applied Treatment and Procedures				
**The most advanced respiratory support applied during hospitalization** **n/N (% of risk category)** **(N = 2179)**				<0.0001	<0.0001 ^a,b^
No oxygen	770/1447 (53.21%)	227/611 (37.15%)	34/121 (28.1%)		
High-flow nasal cannula (non-invasive ventilation)	73/1447 (5.04%)	44/611 (7.2%)	14/121 (11.57%)		0.2601 ^c^
Invasive ventilation	128/1447 (8.85%)	71/611 (11.62%)	13/121 (10.74%)		
Oxygenation parameters from the period of qualification for advanced respiratory support: mean ± SD min–max					
SpO_2_ (N = 630)	90.43 ± 7.98	86.76 ± 9.47	84.73 ± 11.28	<0.0001	<0.0001 ^a^
	50–100	55–99	60–99		
	(420)	(168)	(42)		0.007 ^b^
Respiratory rate, breaths/minute (N = 105)	25.78 ± 8.55	30.89 ± 12.77	30.86 ± 17.32	0.1207	
	13–50	14–72	15–60		0.534 ^c^
	(60)	(38)	(7)		
Duration of mechanical ventilation, days mean ± SD min–max (N = 1386)	1.86 ± 7.31	1.9 ± 6.14	1.09 ± 3.7	0.2342	N/A
	0–91	0–51	0–20		
	−933	−375	−78		
Therapy with catecholamines n/N (% of risk category) (N = 2181)	123/1449 (8.49%)	80/611 (13.09%)	15/121 (12.4%)	0.0042	0.0054 ^a^
					0.5896 ^b^
					1.0 ^c^
Coronary angiography n/N (% of risk category) (N = 2181)	9/1449 (0.62%)	11/611 (1.8%)	10/121 (8.26%)	<0.0001	0.07 ^a^
					<0.0001 ^b^
					0.0022 ^c^
Coronary revascularization n/N (% of risk category) (N = 2181)	8/1449 (0.55%)	10/611 (1.64%)	8/121 (6.61%)	<0.0001	0.1015 ^a^
					<0.0001 ^b^
					0.0136 ^c^
Hemodialysis n/N (% of risk category) (N = 2181)	38/1449 (2.62%)	25/611 (4.09%)	8/121 (6.61%)	0.0225	0.2758 ^a^
					0.064 ^b^
					0.6909 ^c^

Continuous variables are presented as mean ± SD, range (minimum -maximum), and the number of non-missing values. Categorized variables are presented as a number with a percentage. Information about the numbers with valid values is provided in the left column. Abbreviations: N—valid measurements, n—number of patients with parameters above the cut-off point, SD—standard deviation, N/A—non-applicable, OMNIBUS—overall Welch ANOVA or chi-square test, ^a^—low risk vs. medium risk, ^b^—low risk vs. high risk, ^c^—medium risk vs. high risk, W—Welch ANOVA, X—chi-square test.

**Table 5:** Total and in-hospital all-cause mortality in the CHA_2_DS_2_-VASc risk strata.

Variables, Units (N)	Low Risk	Medium Risk	High Risk	Overall Chi-Square Test *p* Value	*p* Value (for Post Hoc Analysis)
	All-Cause Mortality Rate				
**In-hospital mortality n/N (% of risk category)** **(N = 2181)**	**123/1449 (8.49%)**	**160/611 (26.19%)**	**43/121 (35.54%)**	<0.0001	<0.0001 ^a,b^
					0.1404 ^c^
3-month mortality n/N (% of risk category) (N = 2085)	226/1371 (16.48%)	254/594 (42.76%)	66/120 (55.0%)	<0.0001	<0.0001^a,b^
					0.0551 ^c^
6-month mortality n/N (% of risk category) (N = 1113)	238/606 (39.27%)	267/412 (64.65%)	72/95 (75.79%)	<0.0001	<0.0001^a,b^
					0.151 ^c^

Categorized variables are presented as a number with a percentage. Abbreviations: N—valid measurements, n—number of patients with parameters above the cut-off point, SD—standard deviation, ^a^—low risk vs. medium risk, ^b^—low risk vs. high risk, ^c^—medium risk vs. high risk.

**Table 6:** Clinical non-fatal events and hospitalization outcomes in the CHA_2_DS_2_-VASc risk strata.

Variables, Units (N)	Low Risk	Medium Risk	High Risk	OMNIBUS *p* Value	*p* Value (for Post Hoc Analysis)
	Hospitalization				
**Duration of hospitalization, days** **mean ± SD** **min–max** **(N = 2181)**	**10.99 ± 13.3**	**15.11 ± 14.89**	**16.35 ± 15.37**	<0.0001	<0.0001 ^a^
	1–131	1–121	1–87		0.0009 ^b^
	(1449)	(611)	(121)		0.697 ^c^
Admission at ICU n/N (% of risk category) (N = 2181)	135/1449 (9.32%)	68/611 (11.13%)	12/121 (9.92%)	0.4517	N/A
End of hospitalization/N (% of risk category) (N = 2181)				<0.0001	
			
death	125/1449 (8.49%)	160/611 (26.19%)	43/121 (35.54)		<0.0001 ^a,b^
Discharge home—full recovery	1000/1449 (69.01%)	273/611 (44.68%)	42/121 (34.71)		
Transfer to another hospital—worsening	159/1449 (10.97%)	100/611 (16.37%)	20/121 (16.53%)		0.4096 ^c^
Transfer to another hospital—in recovery	167/1449 (11.53)	78/611 (12.77%)	16/121 (13.22%)		
Clinical events					
Aborted cardiac arrest n/N (% of risk category) (N = 2181)	15/1449 (1.04%)	5/611 (0.82%)	4/121 (3.31%)	0.0806	N/A
Shock n/N (% of risk category) (N = 2181)	102/1449 (7.04)	74/611 (12.11%)	11/121 (9.09)	0.0008	0.0007 ^a^
					1.0 ^b,c^
Hypovolemic shock	20/1449 (1.38%)	14/611 (2.29%)	1/121 (0.83%)	0.302	N/A
Cardiogenic shock	10/1449 (0.69%)	15/611 (2.45%)	7/121 (5.79%)	<0.0001	0.0048 ^a^
					0.0004 ^b^
					0.2192 ^c^
Septic shock	75/1449 (5.18%)	57/611 (9.33%)	8/121 (6.61%)	0.0021	0.0019 ^a^
					1.0 ^b,c^
Venous thromboembolic disease n/N (% of risk category) (N = 2181)	47/1449 (3.24%)	19/611 (3.11%)	3/121 (2.48%)	1.00	N/A
Pulmonary embolism n/N (% of risk category) (N = 2181)	38/1449 (2.62%)	18/611 (2.95%)	3/121 (2.48%)	0.8603	N/A
Deep vein thrombosis n/N (% of risk category) (N = 2181)	17/1449 (1.17%)	4/611 (0.65%)	0/121 (0%)		
MI n/N (% of risk category) (N = 2081)	5/1449 (0.35%)	14/611 (2.29%)	7/121 (5.79%)	<0.0001	0.0003 ^a^
					<0.0001 ^b^
					0.1946 ^c^
Acute HF n/N (% of risk category) (N = 2181)	14/1449 (0.97%)	40/611 (6.55%)	22/121 (18.18%)	<0.0001	<0.0001^a,b^
					0.0004 ^c^
Stroke/TIA n/N (% of risk category) (N = 2181)	7/1449 (0.48%)	24/611 (3.93%)	12/121 (9.92%)	<0.0001	<0.0001 ^a,b^
					0.03 ^c^
New cognitive signs and symptoms n/N (% of risk category) (N = 2181)	36/1449 (2.48%)	60/611 (9.82%)	24/121 (19.83%)	<0.0001	<0.0001^a,b^
					0.0081 ^c^
Pneumonia n/N (% of risk category) (N = 2181)	623/1449 (43.0%)	360/611 (58.92%)	77/121 (63.64%)	<0.0001	<0.0001 ^a,b^
					0.1 ^c^
SIRS n/N (% of risk category) (N = 2112)	147/1381 (10.64%)	56/610 (9.18%)	17/121 (14.05%)	0.2482	N/A
Sepsis n/N (% of risk category) (N = 883)	7/586 (1.19%)	15/243 (6.17%)	1/54 (1.85%)	0.0004	0.0005 ^a^
					1.0 ^b^
					0.9617 ^c^
Acute kidney injury n/N (% of risk category) (N = 2181)	102/1449 (7.04%)	105/611 (17.18%)	29/121 (23.97%)	<0.0001	<0.0001 ^a,b^
					0.3069 ^c^
Acute liver dysfunction n/N (% of risk category) (N = 1972)	30/1283 (2.34%)	31/573 (5.41%)	5/116 (4.31%)	0.0024	0.0031 ^a^
					0.6138 ^b^
					1.0 ^c^
MODS n/N (% of risk category) (N = 2181)	22/1449 (1.52%)	11/611 (1.8%)	4/121 (3.31%)	0.2827	N/A
LA n/N (% of risk category) (N = 245)	8/105 (7.62%)	9/103 (8.74%)	5/37 (13.51%)	0.5431	N/A
Hyperlactemia n/N (% of risk category) (N = 245)	81/105 (77.14%)	61/103 (59.22%)	25/37 (67.57%)	0.0213	0.0258 ^a^
					1.0 ^b,c^
Bleedings n/N (% of risk category) (N = 2181)	58/1449 (4.0%)	46/611 (7.53%)	10/121 (8.26%)	0.0014	0.0037 ^a^
					0.1431 ^b^
					1.0 ^c^
Intracranial bleeding n/N (% of risk category) (N = 2181)	7/1449 (0.45%)	12/611 (1.96%)	2/121 (1.65%)	0.0049	0.0109 ^a^
					0.4458 ^b^
					1.0 ^c^
Respiratory tract bleeding n/N (% of risk category) (N = 2181)	20/1449 (1.38%)	11/611 (1.8%)	3/121 (2.48%)	0.4718	N/A
Gastrointestinal tract bleeding n/N (% of risk category) (N = 1047)	20/1449 (1.38%)	20/611 (3.27%)	1/121 (0.83%)	0.0082	0.015 ^a^
					1.0 ^b^
					0.2092 ^c^
Urinary tract bleeding n/N (% of risk category) (N = 1047)	6/1449 (0.41%)	8/611 (1.31%)	4/121 (3.35%)	0.0019	0.1083 ^a^
					0.0147 ^b^
					0.3617 ^c^

Continuous variables are presented as mean ± SD, range (minimum–maximum), and the number of non-missing values. Categorized variables are presented as a number with a percentage. Abbreviations: N—valid measurements, n—number of patients with parameters above the cut-off point, SD—standard deviation, ANOVA—analysis of variance, ICU—intensive care unit, MI—myocardial infarction, HF—heart failure, TIA—transient ischemic attack, SIRS—systemic inflammatory response syndrome, MODS—multiple organ dysfunction syndrome, LA—lactic acidosis, N/A—non-applicable, OMNIBUS—overall Welch ANOVA or chi-square test, ^a^—low risk vs. medium risk, ^b^—low risk vs. high risk, ^c^—medium risk vs. high risk, W—Welch ANOVA, X—chi-square test.

## Data Availability

The original contributions presented in the study are included in the article/Supplementary Material, further inquiries can be directed to the corresponding author.

## Supplementary Materials

The following supporting information can be downloaded at https://www.mdpi.com/article/10.3390/microorganisms12102060/s1, Table S1: LOGRANK_CHA2DS2VASc test statistic; Table S2: Baseline characteristics of the study cohort—treatment applied before hospitalization; Table S3: Laboratory parameters measured during the hospitalisation in the studied cohort.
