# Supplementary material for: Usefulness of the CHA2DS2-VASc Score in Predicting the Outcome in Subjects Hospitalized with COVID-19—A Subanalysis of the COLOS Study
Source: Microorganisms. 2024 Oct 13;12(10):2060. doi: 10.3390/microorganisms12102060 (PMC11510264; doi:10.3390/microorganisms12102060)
Supplement: Supplementary file 1 [file microorganisms-12-02060-s001.zip › microorganisms-3189949-supplementary.pdf]

Table S1. LOGRANK_CHA2DS2VASc test statistic.

	**j = 1**	**j = 2**	**j = 3**	**j = 4**	**j = 5**	**j = 6**	**j = 7**	**j = 8**	**j = 9**
i = 1		206,1341	254,1476	196,6129	164,1123	129,8659	73,28451	51,93191	46,94362
i = 2			**270,1802**	256,6272	256,8149	247,5945	218,0633	208,3805	205,9969
i = 3				261,2534	272,2674	**273,5209**	258,0141	253,8553	252,7725
i = 4					196,6595	199,506	188,3053	185,9535	185,3422
i = 5						146,829	142,2883	141,9339	141,8956
i = 6							98,63321	98,45002	98,65507
i = 7								32,16719	32,32279
i = 8									6,508364

Table S2. Baseline characteristics of the study cohort - treatment applied before hospitalization.

**Variables, units (N)**	**Low risk**	**Medium risk**	**High risk**	Overall Chi-squared test *p value*	*p value* (for post-hoc analysis)
	**Treatment applied before hospitalization**				
**ACEI n/N (% of risk category) (N=2181)**	**98/1449 (6.76%)**	**198/611 (32.41%)**	**55/121 (45,45%)**	<0.0001 [X]	<0.0001 [a, b,] 0.0239 [c]
ARBs n/N (% of risk category) (N=2181)	69/1449 (4.76%)	65/611 (10.64%)	10/121 (8.26%)	<0.0001 [X]	<0.0001 [a] 0.4192 [b] 1.0 [c]
MRAs n/N (% of risk category) (N=2181)	17/1449 (1.17%)	61/611 (9.98%)	22/121 (18.18%)	<0.0001 [X]	<0.0001 [a,b] 0.0439 [c]
Sacubitril/valsartan n/N (% of risk category) (N=2181)	6/1449 (0.41%)	4/611 (0.65%)	0/121 (0%)	0.7151 [X]	N/A
β-blocker n/N (% of risk category) (N=2181)	187/1449(12.91%)	273/611 (44.68%)	72/121 (59.5%)	<0.0001 [X]	<0.0001 [a, b] 0.0118 [c]
Digitalis glycoside n/N (% of risk category) (N=2181)	3/1449 (0.21%)	13/611 (2.13%)	3/121 (2.48%)	<0.0001 [X]	<0.0001 [a] 0.0226 [b] 1.0 [c]
Calcium channel blocker (non-dihydropiridines) n/N (% of risk category) (N=2181)	12/1449 (0.83%)	22/611 (3.6%)	3/121 (3.48%)	<0.0001 [X]	<0.0001[a] 0.3084 [b] 1.0 [c]
Calcium channel blocker (dihydropiridines) n/N (% of risk category) (N=2181)	89/1449 (6.14%)	132/ 611 (21.6%)	40/121 (33.06%)	<0.0001 [X]	<0.0001 [a, b] 0.0282 [c]
α-adrenergic blocker n/N (% of risk category) (N=2181)	40/1449 (2.76%)	58/611 (9.49%)	20/121 (16.53%)	<0.0001 [X]	<0.0001[a, b] 0.0994 [c]
Thiazide or thiazide-like diuretic	57/1449 (3.93%)	77/611 (12.6%)	15/121 (12.4%)	<0.0001 [X]	<0.0001 [a]

n/N (% of risk category) (N=2181)					0.0002 [b]<br>1.0 [c]
Loop diuretic n/N (% of risk category) (N=2181)	38/1449(2.62 %)	103/611 (11.86%)	44/121 (36.36%)	<0.0001 [X]	<0.0001 a, [b, c]
Statin n/N (% of risk category) (N=2181)	85/1449 (5.87%)	198/611 (32.41%)	66/121 (54.54%)	<0.0001 [X]	<0.0001 [a, b, c]
Acetylsalicylic acid n/N (% of risk category) (N=2181)	71/1449 (4.9%)	146/611 (23.9%)	40/121 (33.06%)	<0.0001 [X]	<0.0001 [a, b]<br>0.1362 [c]
The second antiplatelet drug - P2Y12 inhibitor n/N (% of risk category) (N=2181)	2/1449 (0.14%)	22/611 (3.6%)	15/121 (12.4%)	<0.0001 [X]	<0.0001 [a, b]<br>0.0009 [c]
LMWH n/N (% of risk category) (N=2181)	69/1449 (4.76%)	59/611 (9.66%)	13/121 (10.74%)	<0.0001 [X]	0.0001[a]<br>0.0257 [b]<br>1.0 [c]
VKA n/N (% of risk category) (N=2181)	10/1449 (0.69%)	31/611 (5.07%)	6/121 (4.96%)	<0.0001 [X]	<0.0001[a]<br>0.0023 [b]<br>1.0 [c]
NAOC n/N (% of risk category) (N=2181)	16/1449 (1.1%)	63/611 (10.31%)	27/121 (22.31%)	<0.0001 [X]	<0.0001[a, b]<br>0.0013 [c]
Insulin n/N (% of risk category) (N=2181)	38/1449 (2.62%)	64/611 (10.47%)	29/121 (23.97%)	<0.0001 [X]	<0.0001 [a, b]<br>0.0003 [c]
Metformin n/N (% of risk category) (N=2181)	57/1449 (3.93%)	129/611 (21.11%)	35/121 (28.93%)	<0.0001 [X]	<0.0001 [a, b]<br>0.2333 [c]
SGLT2 inhibitor n/N (% of risk category) (N=2181)	6/1449 (0.41%)	16/611 (2.62%)	5/121 (4.13%)	<0.0001 [X]	0.0001 [a]<br>0.0024 [b]<br>1.0 [c]
Oral antidiabetics other than SGLT2 inhibitor and metformin n/N (% of risk category) (N=2181)	8/1449 (0.55%)	59/611 (9.66%)	22/121 (18.18%)	<0.0001 [X]	<0.0001 [a, b]<br>0.0312 [c]
Proton pump inhibitor n/N (% of risk category) (N=2181)	82/1449 (5.66%)	132/611 (21.6%)	36/121 (29.75%)	<0.0001 [X]	<0.0001 [a, b]<br>0.2022 [c]
Oral corticosteroid n/N (% of risk category) (N=2181)	66/1449 (4.55%)	22/611 (3.6%)	4/121 (3.31%)	0.54 [X]	N/A
Immunosuppression other than oral corticosteroid n/N (% of risk category) (N=2181)	53/1449 (3.66%)	18/611 (2.95%)	2/121 (1.65%)	0.4906 [X]	N/A

Categorized variables are presented as a number with a percentage. Information about the numbers with valid values is provided in the left column. Abbreviations: N-valid measurements. n - number of patients with parameter above cut-off point. ACEI - angiotensin-converting-enzyme inhibitors. ARBs- angiotensin receptor blockers. MRAs - mineralocorticoid receptor antagonists LMWH –low molecular weight heparyn. VKA- vitamin K antagonists. NOAC - new oral anticoagulants. SGLT2 inhibitors – sodium glucose co-transporter-2 inhibitors. N/A – not-applicable. a – low risk vs. medium risk. b – low risk vs. high risk. c – medium risk vs. high risk stratum, X – Chi-square test.

Table S3. Laboratory parameters measured during the hospitalisation in the studied cohort.

Parameters	Time of assessment	Units	Low risk	Medium risk	High risk	OMNIBUS *p value*	*p value* (for post-hoc analysis)
Complete Blood Count (CBC)							
Leucocytes median (Q1-Q3) min-max (N=2046)	On admission	10^3/µl	7,275 (5,21-10,12)<br>0.51-304.02<br>(1330)	7,55 (5,53- 10,64)<br>0.56-150.93<br>(596)	7,53 (5,72-10,53)<br>1.83-215.97<br>(120)	0.4365 [W]	N/A
	On discharge		7,65 (5,65-10,06)<br>0.51-342.01<br>(1330)	5,72 (7,99-11,06)<br>0.44-58.49<br>(596)	7,84 (5,80-11,92)<br>1.31-314.44<br>(120)	0.0327 [W]	0.032 [a]<br>0.601 [b]<br>0.849 [c]
Lymphocytes median (Q1-Q3) min-max (N=1294)	On admission	10^3/µl	0,99 (0,69-1,51)<br>0.03-296.61<br>(767)	0,94 (0,63-1,38)<br>0.09-78.58<br>(438)	0,9 (0,55-1,33)<br>0.12-3.8<br>(89)	0.0596 [W]	N/A
	On discharge		1,56 (1,03-2,16)<br>0.06-114.12<br>(767)	1,19 (0,78-1,73)<br>0.05-66.97<br>(438)	0,9 (0,58-1,4)<br>0.14-3.26<br>(89)	<0.0001 [W]	0.16 [a]<br><0.0001[b]<br>0.015 [c]
Haemoglobin mean±SD min-max (N=2046)	On admission	g/dL	13.21±2.19<br>3.9-20.3<br>(1330)	12.45±2.33<br>5.3-18.9<br>(596)	12.24±2.51<br>6.1-18.8<br>(120)	<0.0001 [W]	<0.0001[a]<br>0.0002 [b]<br>0.664 [c]
	On discharge		12.73±2.22<br>4.5-18.7<br>(1330)	11.84±2.34<br>5.4-18.9<br>(596)	11.61±2.26<br>5.5-17.7<br>(120)	<0.0001 [W]	<0.0001[a, b]<br>0.571 [c]
Platelets mean±SD min-max (N=2046)	On admission	10^3 /µl	233.73±110.23<br>3-1356<br>(1330)	229.86±106.9<br>0-735<br>(596)	223.33±88.47<br>85-518<br>(120)	0.4288 [W]	N/A
(N=2044)	On discharge		269.5±130.09<br>2-1101<br>(1330)	238.84±112.92<br>4-675<br>(596)	217.71±96.18<br>41-508<br>(120)	<0.0001 [W]	0.001 [a,b]<br>0.087 [c]
Acid-base balance in the arterial blood gas							
PH mean±SD min-max (N=276)	On admission		7.43±0.08<br>7.04-7.58<br>(121)	7.42±0.07<br>7.09-7.54<br>(115)	7.42±0.07<br>7.25-7.53<br>(40)	0.7278 [W]	N/A
PaO$_2$	On	<60 mmHg	37/121	44/115 (38.26%)	20/40	0.877 [X]	N/A

n/N (% of risk category)	admission	respiratory insufficiency	(30.58%)		(50.0%)		
		≥60 mmHg	84/121 (69.42%)	71/115 (61.74%)	20/40 (50.0%)		
mean±SD min-max (N=276)			72.05±26.52 12.8-100 (121)	74.35±40.46 26.8-100 (115)	73.02±49.51 23.7-100 (40)	0.8821 [W]	N/A
$PaCO_2$ n/N (% of risk category)	On admission	≥ 45 mmHg hypercapnia	22/121 (18.18%)	17/115 (14.78%)	6/40 (15.0%)	0.7567	N/A
		< 45 mmHg	99/121 (81.82%)	98/115 (85.22%)	34/40 (85.0%)		
mean±SD min-max (N=276)			38.16±10.9 20.2-82.4 (121)	37.12±9.18 19.7-74.9 (115)	38.22 ±10.71 25.7-88.4 (40)	0.6873 [W]	N/A
HCO_3 standard mean±SD min-max (N=272)	On admission	mmol/L	24.89±3.58 12.5-32.9 (120)	24.2±4.62 12.1-39.5 (113)	24.55±4.04 13.5-32.3 (39)	0.4527 [W]	N/A
BE median (Q1-Q3) min-max (N=108)	On admission		1.8 ((-)1.3-3.7) (-)15.7–10.5 (38)	1.2((-)0.4-4.55) (-)12.5-15.7 (53)	1.35((-)1.53-4.83) (-)3.7–8.0 (17)	0.5292 [W]	N/A
Lactates mean±SD min-max (N=245)	On admission		2.26±0.95 0.6-5.9 (105)	2.4±1.96 0.5-12.8 (103)	2.32±1.16 0.5-5.6 (37)	0.7775 [W]	N/A
Electrolytes. Inflammatory and iron biomarkers							
Na mean±SD min-max (N=2028)	On admission	mmol/L	138.24±4.61 101-159 (1316)	137.72±6.95 105-175 (592)	138.34±5.37 126-163 (120)	0.2329 [W]	N/A
K mean±SD min-max (N=2035)	On admission	mmol/L	4.06±0.57 2.4-7.03 (1321)	4.18±0.74 2.0-7.5 (594)	4.25±0.89 2.6-8.7 (120)	0.0006 [W]	0.002 [a] 0.071 [b] 0.708 [c]
CRP median (Q1-Q3) min-max (N=2016)	On admission	mg/L	45.81(11.3-113.6) 0.13-538.55 (1304)	58.27(17.9-119.61) 0.32-487.38 (592)	50.24(13.07-105) 0.4-431.65 (120)	0.5044 [W]	N/A
Procalcitonin median (Q1-Q3) min-max (N=1472)	On admission	ng/mL	0.08 (0.03-0.215) 0.01-51.18 (925)	0.12 (0.05-0.48) 0.01-196.04 (449)	0.16 (0.06-0.575) 0.01-27.59 (98)	0.0183 [W]	0.023 [a] 0.437 [b] 0.271 [c]
IL-6 median (Q1-Q3) min-max	On admission	pg/mL	15.25 (5.46-41.07) 2-9099	24 (7.93-54.8) 2-1000	26 (9.12-59.55) 3.97-421	0.85 [W]	N/A

(N=701)			(484)	(182)	(35)		
D-dimer<br>median (Q1-Q3)<br>min-max<br>(N=1577)	On admission	µg/L	0.95 (0.56-1.67)<br>0.15-132.82<br>(1014)	1.4 (0.89-3.78)<br>0.2-128.0<br>(473)	1.27 (0.74-2.52)<br>0.22-128.0<br>(90)	0.1459 [W]	N/A
Protrombin rate<br>mean±SD<br>min-max<br>(N=1921)	On admission	%	87.6±19.27<br>9-148<br>(1245)	77.65±23.16<br>3-131<br>(562)	72.45±25.37<br>2-124<br>(114)	<0.0001 [W]	<0.0001 [a, b]<br>0.11[c]
INR<br>n/N<br>(% of risk category)<br>(N=1921)	On admission	>1.5	47/1245<br>(3.78%)	76/562 (13.5%)	20/114 (17.54%)	<0.0001 [X]	0.0014 [a]<br><0.0001 [b,c]
APTT<br>n/N<br>(% of risk category)<br>(N=1864)	On admission	>60 s	23/1205<br>(1.91%)	16/548 (2.92%)	7/111 (6.31%)	0.0173 [X]	0.6615 [a]<br>0.03 [b]<br>0.264 [c]
Urea<br>mean±SD<br>min-max<br>(N=1852)	On admission	mg/dL	43.14±36.73<br>5-307<br>(1174)	68.23±51.07<br>11-369<br>(567)	77.16±50.35<br>17-336<br>(114)	<0.0001 [W]	<0.0001 [a, b]<br>0.2 [c]
Creatinine<br>median (Q1-Q3)<br>min-max<br>(N=1959)	On admission	mg/dL	0.88 (0.73-1.1)<br>0.26-14.87<br>(1244)	1.02 (0.79-1.49)<br>0.39-14.77<br>(595)	1.23 (0.95-2.03)<br>0.64-9.27<br>(120)	<0.0001 [W]	<0.0001 [a, b]<br>0.167 [c]
	On discharge		0.26-14.87<br>(1244)	0.4-14.82<br>(595)	0.49-9.27<br>(120)	<0.0001 [W]	<0.0001[a]<br>0.0002 [b]<br>0.578 [c]
eGFR<br>mean±SD<br>min-max<br>(N=1954)	On admission	ml/min/1.73 m^2	84.68±34.04<br>0-180<br>(1239)	59.99±30.11<br>3-180<br>(595)	49.34±26.46<br>6-131<br>(120)	<0.0001 [W]	<0.0001 [a, b]<br>0.0004 [c]
	On discharge		88.16±34.84<br>0-180<br>(1239)	65.06±33.21<br>3-180<br>(595)	58.45±30.32<br>6-154<br>(120)	<0.0001 [W]	<0.0001 [a, b]<br>0.084 [c]
Total protein<br>mean±SD<br>min-max<br>(N=606)	On admission	g/L	6.07±0.89<br>3.5-9.5<br>(334)	5.81±0.8<br>3.4-8.2<br>(206)	5.87±1.0<br>3.3-8.2<br>(56)	0.0024 [W]	0.002 [a]<br>0.331 [b]<br>0.928 [c]
Albumin<br>mean±SD<br>min-max<br>(N=663)	On admission	g/L	3.18±0.6<br>1.5-5.1<br>(376)	3.01±0.56<br>0.7-4.4<br>(229)	3.08±0.66<br>1.7-4.9<br>(58)	0.0038 [W]	0.002 [a]<br>0.576 [b]<br>0.736 [c]
AST<br>median (Q1-Q3)<br>min-max<br>(N=1440)	On admission	IU/L	37.5 (24-65)<br>6-2405<br>(890)	35 (23-61.5)<br>5-4776<br>(453)	37 (25-54.5)<br>11-731<br>(97)	0.3917 [W]	N/A
ALT<br>median (Q1-Q3)<br>min-max<br>(N=1587)	On admission	IU/L	31 (19-56)<br>4-1411<br>(989)	27 (17-45.5)<br>4-3700<br>(493)	23 (16-39)<br>6-612<br>(105)	0.0026 [W]	0.998 [a]<br>0.002 [b]<br>0.082 [c]

median (Q1-Q3)<br>min-max<br>(N=1405)	admission		0.1-19.1<br>(865)	0.1-15.1<br>(447)	0.3-3.6<br>(93)		
LDH<br>mean±SD<br>min-max<br>(N=1231)	On admission	U/L	425.72±371.74<br>50-7100<br>(786)	421.2±521.13<br>44-9505<br>(368)	398.22±268.31<br>141-1863<br>(77)	0.7125 [W]	N/A
Cardiac biomarkers							
BNP<br>median (Q1-Q3)<br>min-max<br>(N=359)	On admission	pg/mL	65 (25.4-169.6)<br>1.7-6924.2<br>(160)	140.1 (63.32-332.77)<br>3-11275.7<br>(155)	406.7 (140.9-739.05)<br>25.6-13368.4<br>(44)	0.0002 [W]	0.004 [a]<br>0.011 [b]<br>0.171 [c]
	On discharge		1.7-6924.2<br>(160)	3-9936.7<br>(155)	23.6-13368.4<br>(44)	0.0006 [W]	0.006 [a]<br>0.026 [b]<br>0.187 [c]
NT-proBNP<br>median (Q1-Q3)<br>min-max<br>(N=379)	On admission	ng/mL	255.3 (90.45-759.27)<br>12-70000<br>(188)	3018.3 (797.3-9243.3)<br>18.2-70000<br>(146)	5556.35 (1994.28-16526.7)<br>360.8-70000<br>(45)	<0.0001 [W]	<0.0001[a]<br>0.001 [b]<br>0.334 [c]
	On discharge		12-70000<br>(188)	18.2-70000<br>(146)	360.8--70000<br>(45)	<0.0001 [W]	<0.0001 [a]<br>0.0004 [b]<br>0.092 [c]
Troponin T<br>normal value:<br>*F < 15.6 pg/ml*<br>*M ≤ 34.2 pg/ml*<br>median (Q1-Q3)<br>min-max<br>(N=1173)	On admission	pg/mL	7.3 (3.1-22.88)<br>0-49161<br>(693)	24.4 (10.4-90.5)<br>1.0-125592.6<br>(390)	48.4 (17.35-149.5)<br>3.3-94365.5<br>(90)	0.0377 [W]	0.078 [a]<br>0.328 [b]<br>0.883 [c]
	On discharge		0.2-174652.6<br>(693)	0.8-109359.5<br>(390)	3.4-94365.5<br>(90)	0.3505 [W]	N/A
LDL-cholesterol.<br>mean±SD<br>min-max<br>(N=448)	On admission	mg/dL	99.63±50.32<br>6-510<br>(232)	85.22±41.9<br>6-215<br>(168)	73.27±44.32<br>14-210<br>(48)	0.0003 [W]	0.006 [a]<br>0.001 [b]<br>0.225 [c]
Hormones							
TSH<br>median (Q1-Q3)<br>(N=819)	On admission	mIU/L	1 (0.51-1.7)<br>0-28.81<br>(462)	0.95 (0.51-1.69)<br>0-38.24<br>(292)	1.21 (0.67-2.43)<br>0-11.16<br>(65)	0.3112 [W]	N/A
fT4 n<br>mean±SD<br>min-max<br>(N=338)	On admission	pmol/L	12.55±2.94<br>5.92-33.47<br>(195)	13.06±3.72<br>7.72-36.6<br>(118)	14.16±3.3<br>8.89-22.42<br>(25)	0.0536 [W]	N/A
fT3<br>mean±SD<br>min-max<br>(N=314)	On admission	pmol/L	2.29±1.9<br>0.95-25.25<br>(183)	1.92±0.83<br>0.95-6.85<br>(109)	1.95±0.75<br>0.95-4.13<br>(22)	0.079 [W]	N/A

Continuous variables are presented as: mean ± SD. range (minimum -maximum) and number of non-missing values. Categorized variables are presented as: a number with a percentage. Information about the numbers with valid values is provided in the left column. Abbreviations: N-valid measurements. n - number of patients with parameter above cut-off point. SD - standard deviation. N/A – non-applicable, OMNIBUS – overall Welch ANOVA or Ch-square test. a – low risk vs. medium risk. b – low risk vs. high risk. c – medium risk vs. high risk,, V – Welch ANOVA, X – Chi-square test.
